# Supplementary material for: Regulation of Immune Checkpoint Antigen CD276 (B7-H3) on Human Placenta-Derived Mesenchymal Stromal Cells in GMP-Compliant Cell Culture Media
Source: Int J Mol Sci. 2023 Nov 16;24(22):16422. doi: 10.3390/ijms242216422 (PMC10671289; doi:10.3390/ijms242216422)
Supplement: Supplementary file 1 [file ijms-24-16422-s001.zip › ijms-2680858-supplementary.pdf]

Figures and legends to the online supplement

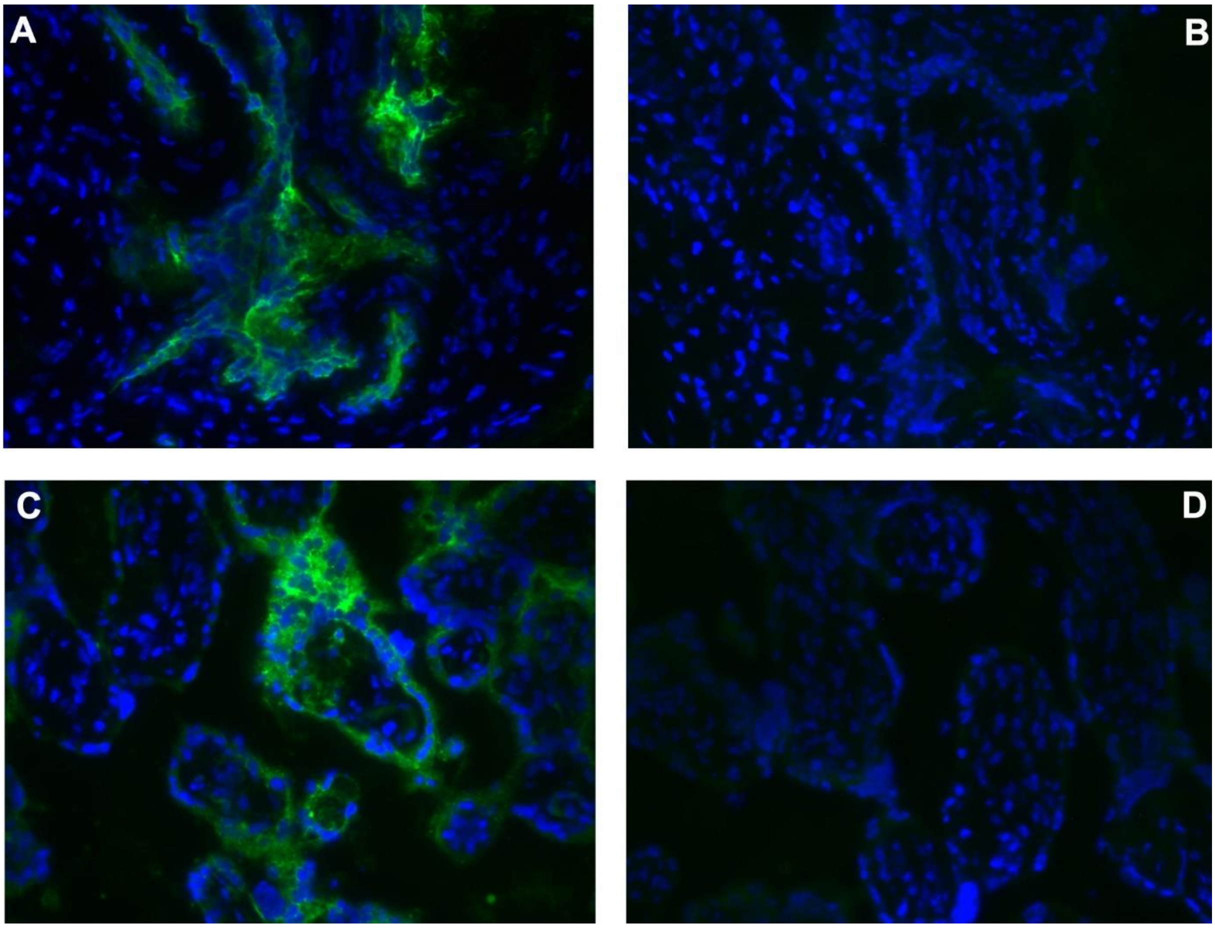

Online supplement Figure S1. Detection of CD73 in human term placenta. Cryosections were generated from tissue samples from the fetal (A, B) and maternal (C, D) tissue samples from the placenta. The cyosections were stained with anti-CD73 antibody followed by AlexaFluor488-labelled detection antibody (A, C), or by detection antibody only (B, D). The slides were counterstained with DAPI to visualize the cell nuclei. All micrographs were taken with a 20x objective. CD73-positive cells were stained in both, fetal and maternal tissue samples of the term placenta.

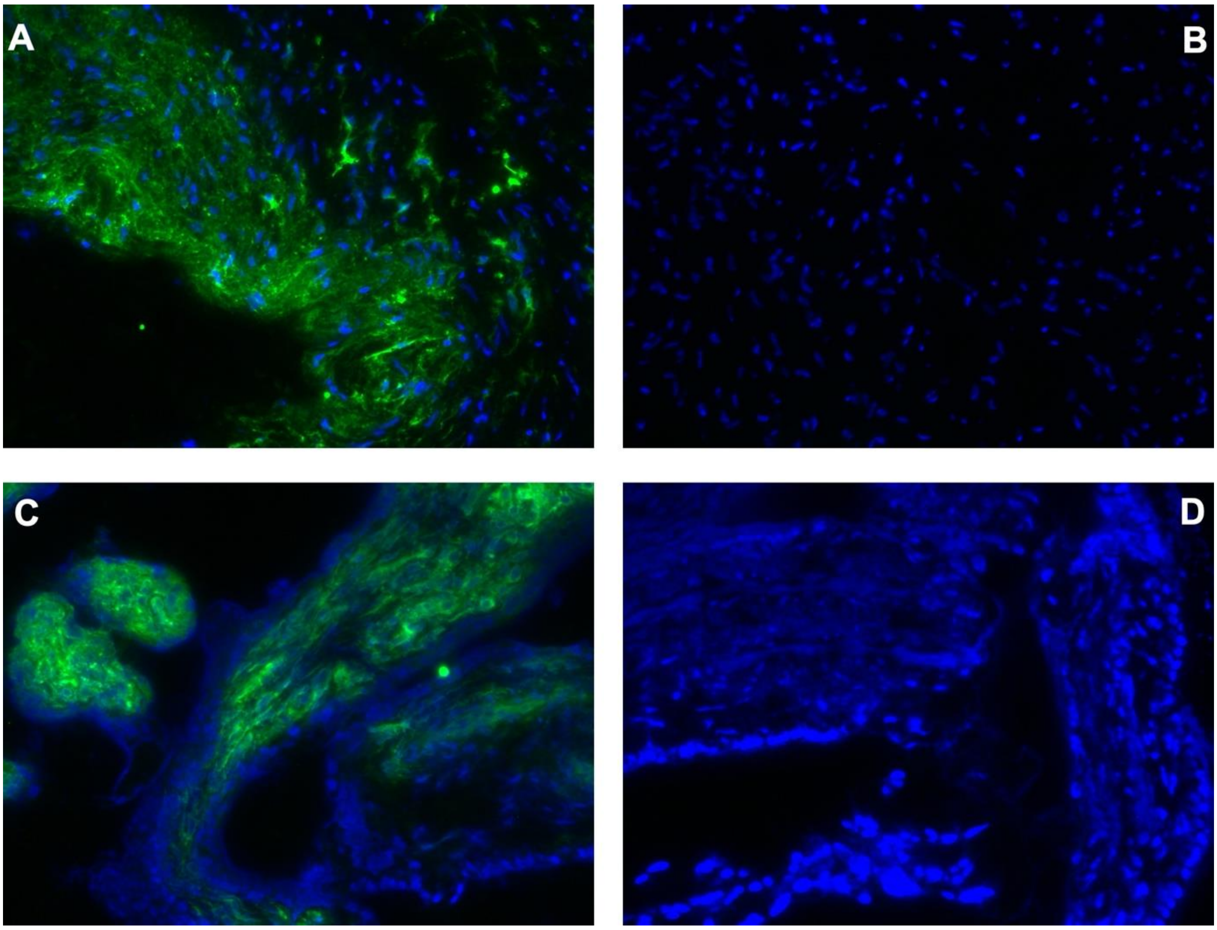

Online supplement Figure S2. Detection of CD90 in human term placenta. Cryosections were generated from tissue samples taken from the fetal (A, B) and maternal (C, D) parts of the placenta. The cryosections were stained with anti-CD105 antibody followed by AlexaFluor488-labelled detection antibody (A, C), or with detection antibody only (B, D). The slides were counterstained with DAPI to visualize the cells nuclei. All micrographs are taken with a 20x objective. Some CD105-positive cells were detected in both, the fetal and maternal tissue samples of the term placenta.

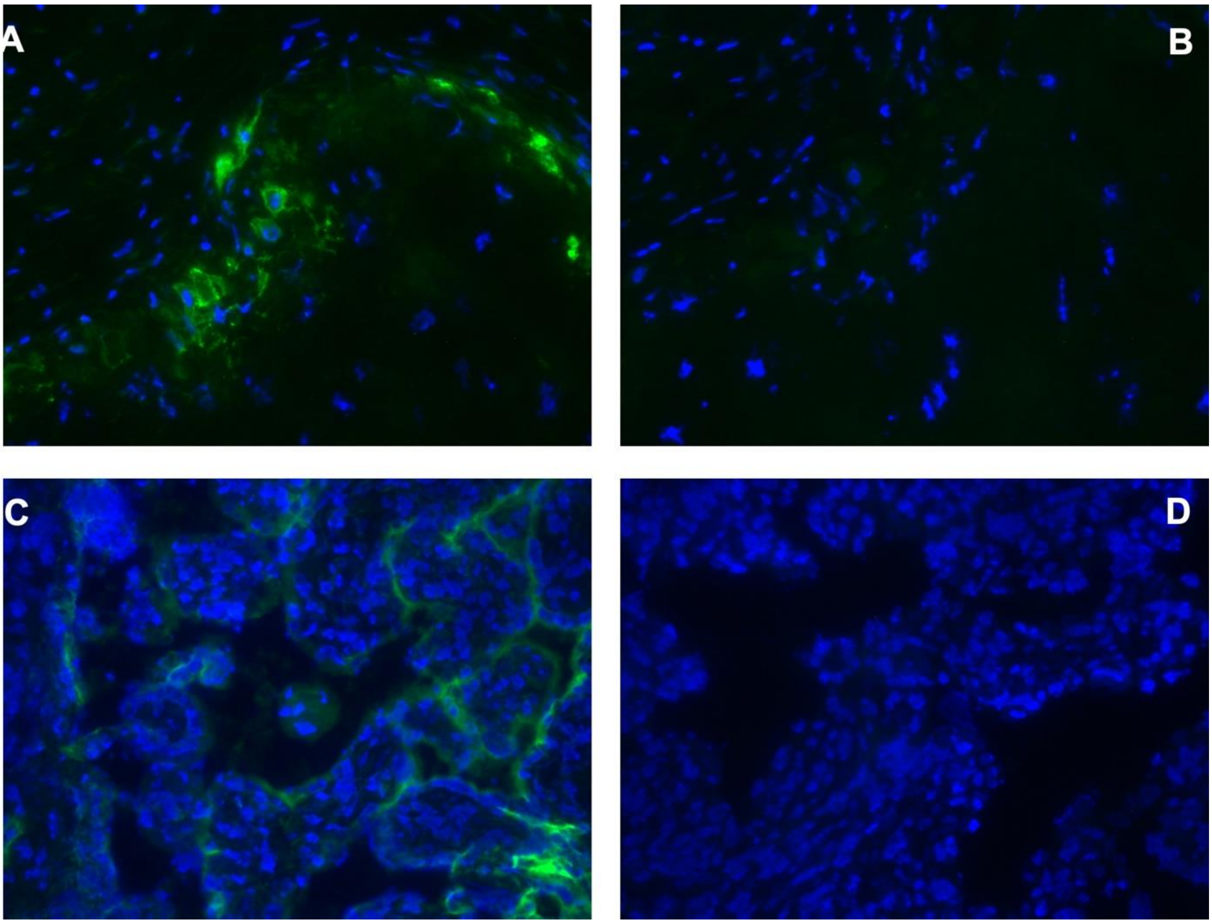

Online supplement Figure S3. Detection of CD105 in human term placenta. Cryosections were generated from tissue samples taken from the fetal part (A, B) and maternal part (C, D) of the placenta. The cyosections were stained with anti-CD90 antibody, followed by AlexaFluor488-labelled detection antibody (A, C), or by detection antibody only (B, D). The sides were counterstained with DAPI to visualize the nuclei of the cells. All micrographs are taken with a 20x objective. CD90-positive cells were detected in both, the fetal and in the maternal part of term placenta.

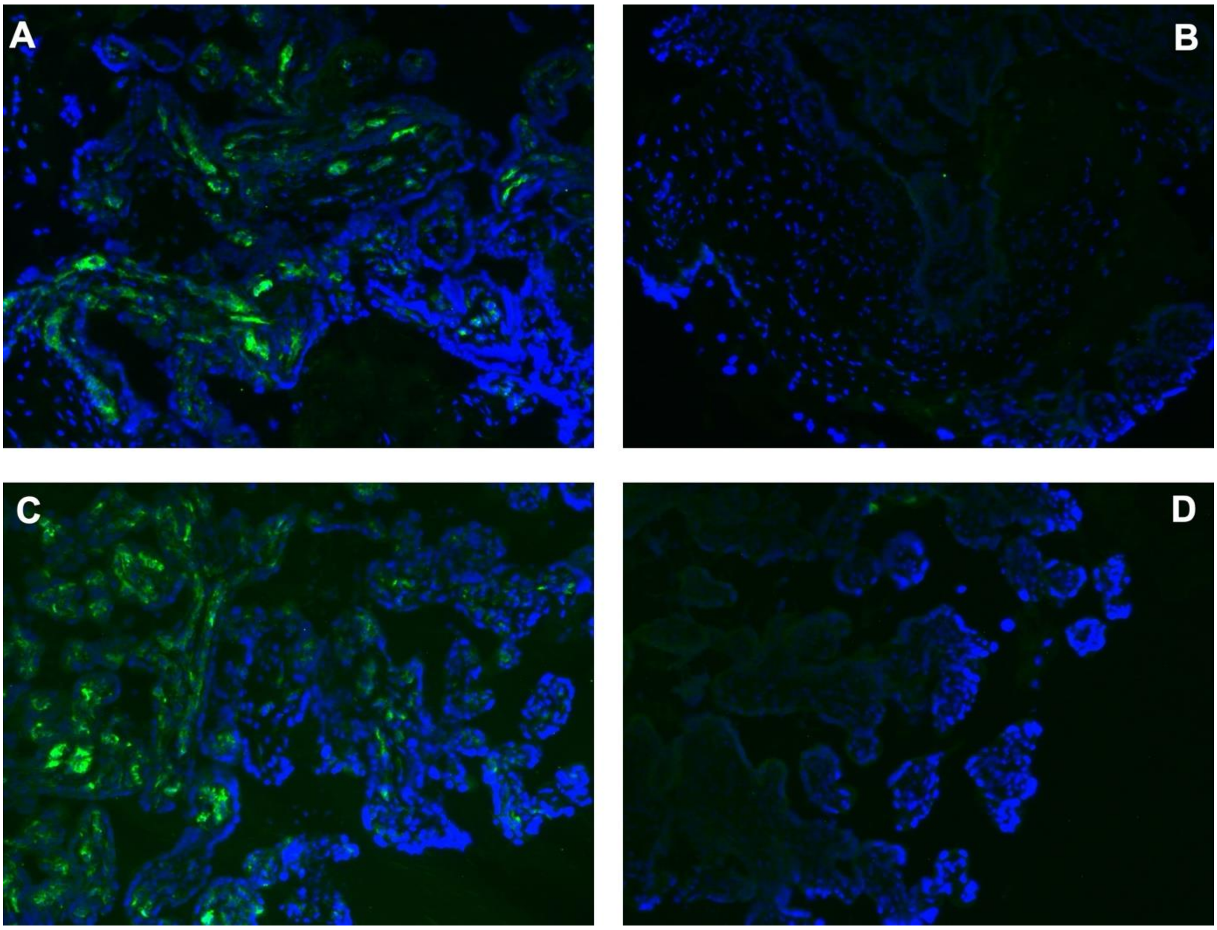

Online supplement Figure S4. Detection of the pericyte marker NG2 in human term placenta. Cryosections were generated from tissue samples taken from the fetal part (A, B) and maternal part (C, D) of the placenta. The cyosections were stained with anti-NG2 antibody followed by AlexaFluor488-labelled detection antibody (A, C), or by detection antibody only (B, D). The sides were counterstained with DAPI to visualize the cells nuclei. All micrographs are taken with a 20x objective. NG2-positive cells were detected in both, the fetal and the maternal tissue samples of the term placenta.

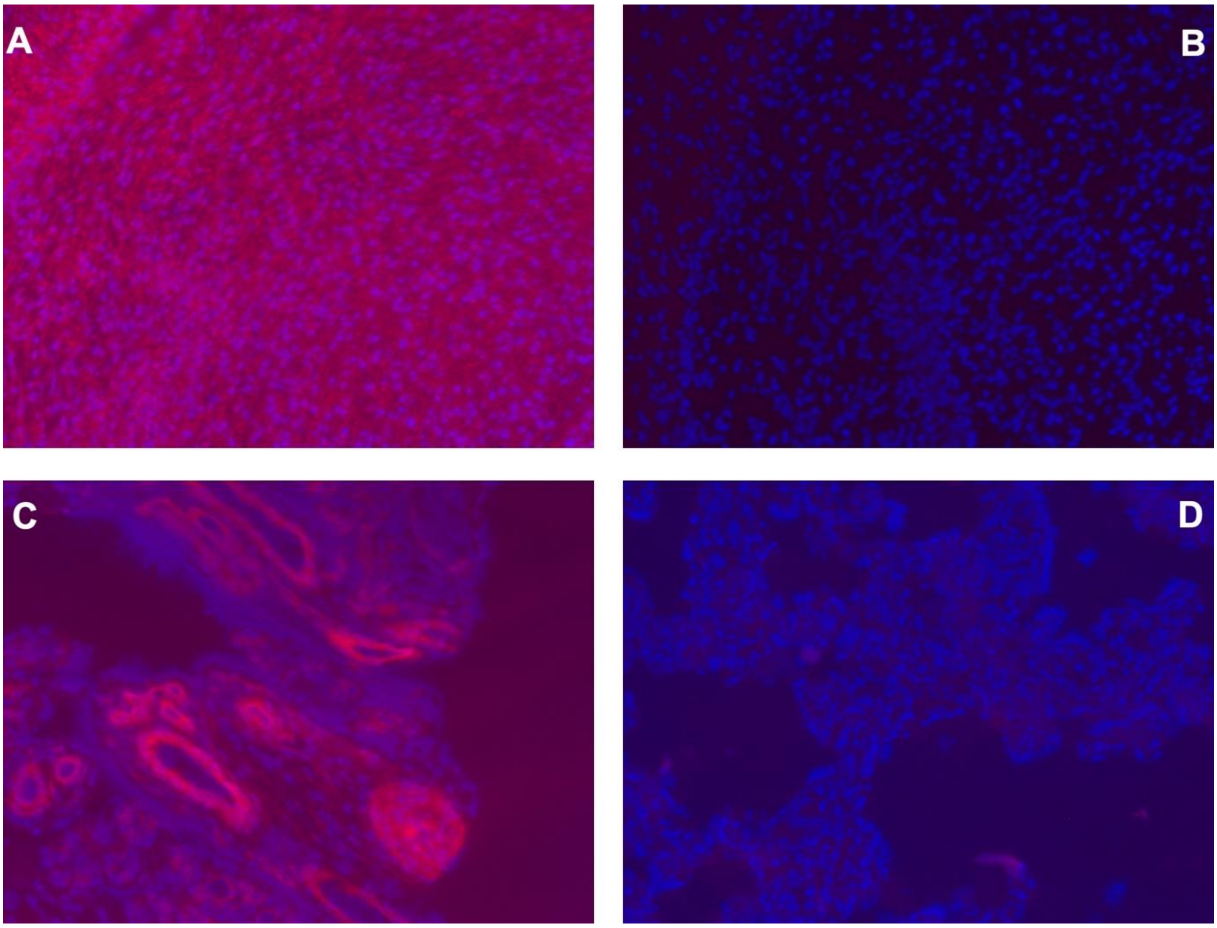

Online supplement Figure S5. Detection of CD146 in human term placenta. Cryosections were generated from tissue samples taken from the fetal part and maternal tissue samples of the placenta. The cyosections were stained with PE-labelled anti-CD146 antibody (A, C), or with Cy-3 labeled goat-anti-rabbit antibody as controls (B, D). The slides were counterstained with DAPI to visualize the nuclei of the cells. All micrographs are taken with a 20x objective. In the fetal part, virtually all cells expressed CD146 while in the maternal part, CD146 was found in the villous zone and around vessels.

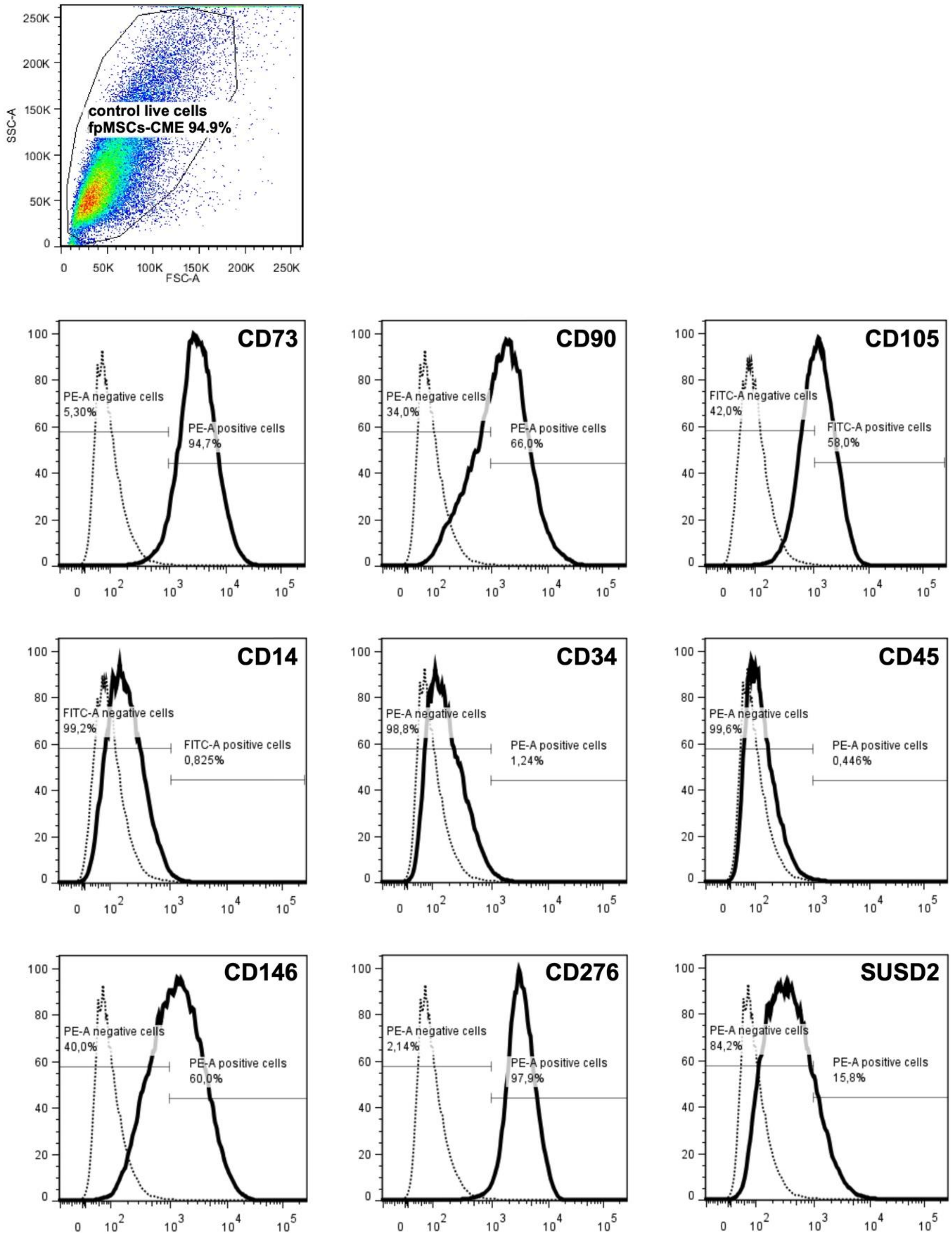

Online supplement Figure S6: Detection of cell surface markers on fpMSCs expanded for two passages in CME-medium. The fpMSCs were expanded to the second passage of *in vitro* culture in CME-medium. The levels of expression of cell surface antigens were investigated by FC. Live cells were gated by determining the side (SSC) and forward (FSC) scatters (upper left panel). The mean fluorescence intensities (x-axes) of the different antigen stainings are presented as percent of

maximum staining (y-axes), as indicated. All fpMSCs expanded in CME-medium expressed the mesenchymal antigens CD73 (94.7%), most expressed CD90 (66%), and CD105 (58%), and less than 1.5% of cells expressed CD14, CD34, or CD45, respectively. The osteogenic marker CD146 (60%) and immune checkpoint antigen CD276 (97.9%) were prominent, and 15.9 % of cells expressed the stem cell marker SUSD2 at a moderate level. The figure shows a representative analysis of cells from one placenta.

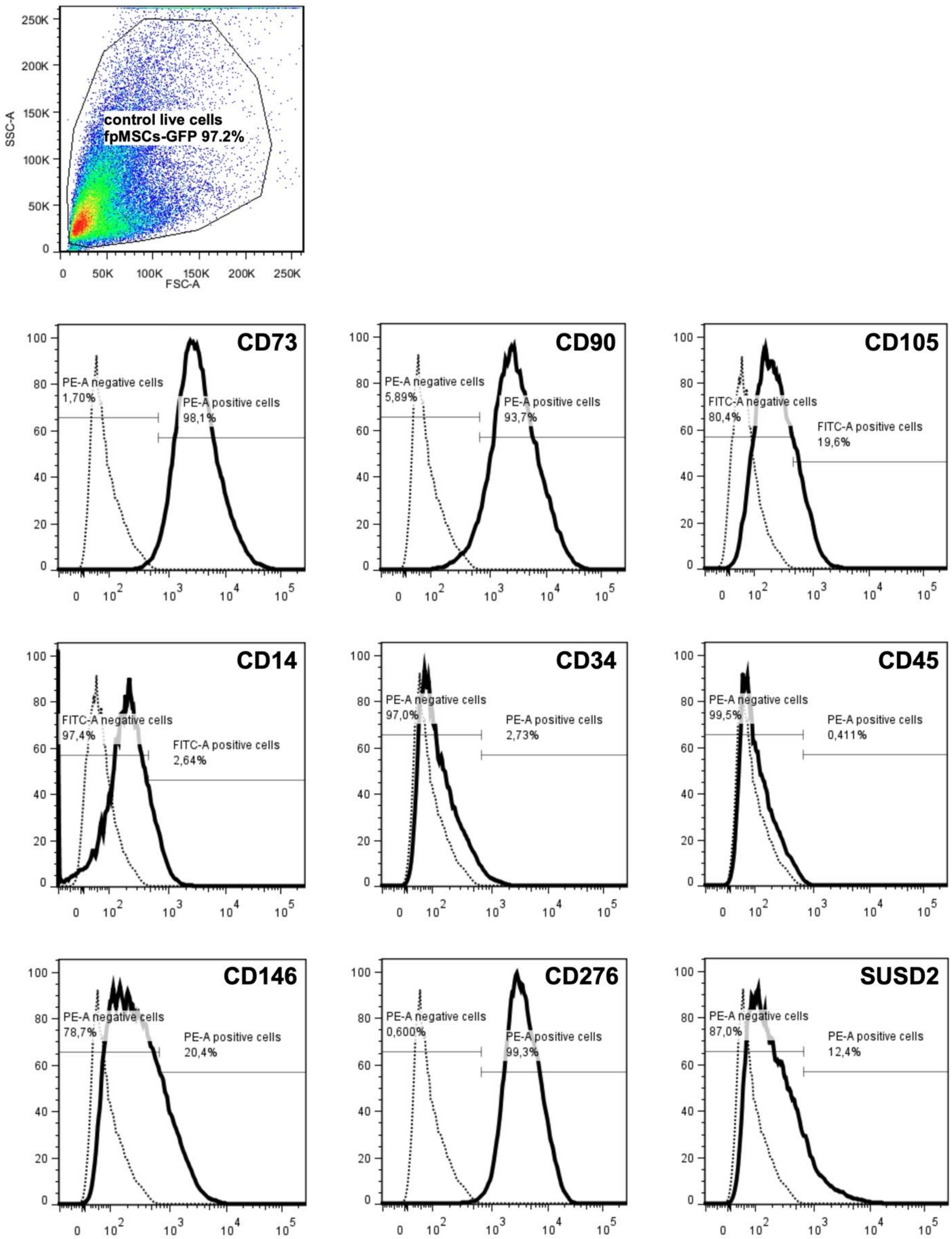

Online supplement Figure S7: Detection of cell surface markers on fpMSCs expanded for two passages in GMP-medium. The fpMSCs were expanded to the second passage of *in vitro* culture in GMP- medium. The levels of expression of cell surface antigens were investigated by FC. Live cells were gated by determining the side (SSC) and forward (FSC) scatters (upper left panel). The mean

fluorescence intensities (x-axes) of the different antigen stainings are presented as percent of maximum staining (y-axes) as indicated. The fpMSCs expanded in GMP-medium expressed the mesenchymal antigens CD73 (98.1%) and CD90 (93.7%) at a prominent level. Only 19.6% of cells expressed CD105, and CD105 staining intensity was low. Little CD14 was found on 2.64% of cells, and 2.73% expressed CD34 with very low staining intensity. Expression of CD45 was not recorded (0.41%). Expression of the osteogenic marker CD146 was moderate on 20.4% of cells. The immune checkpoint antigen CD276 was expressed on all fpMSCs (99.3%) with prominent staining intensity. The stem cell marker SUSD2 was expressed moderately on a few cells (12.4%). The figure shows a representative analysis of cells from the same placenta as presented in os Fig. 6.

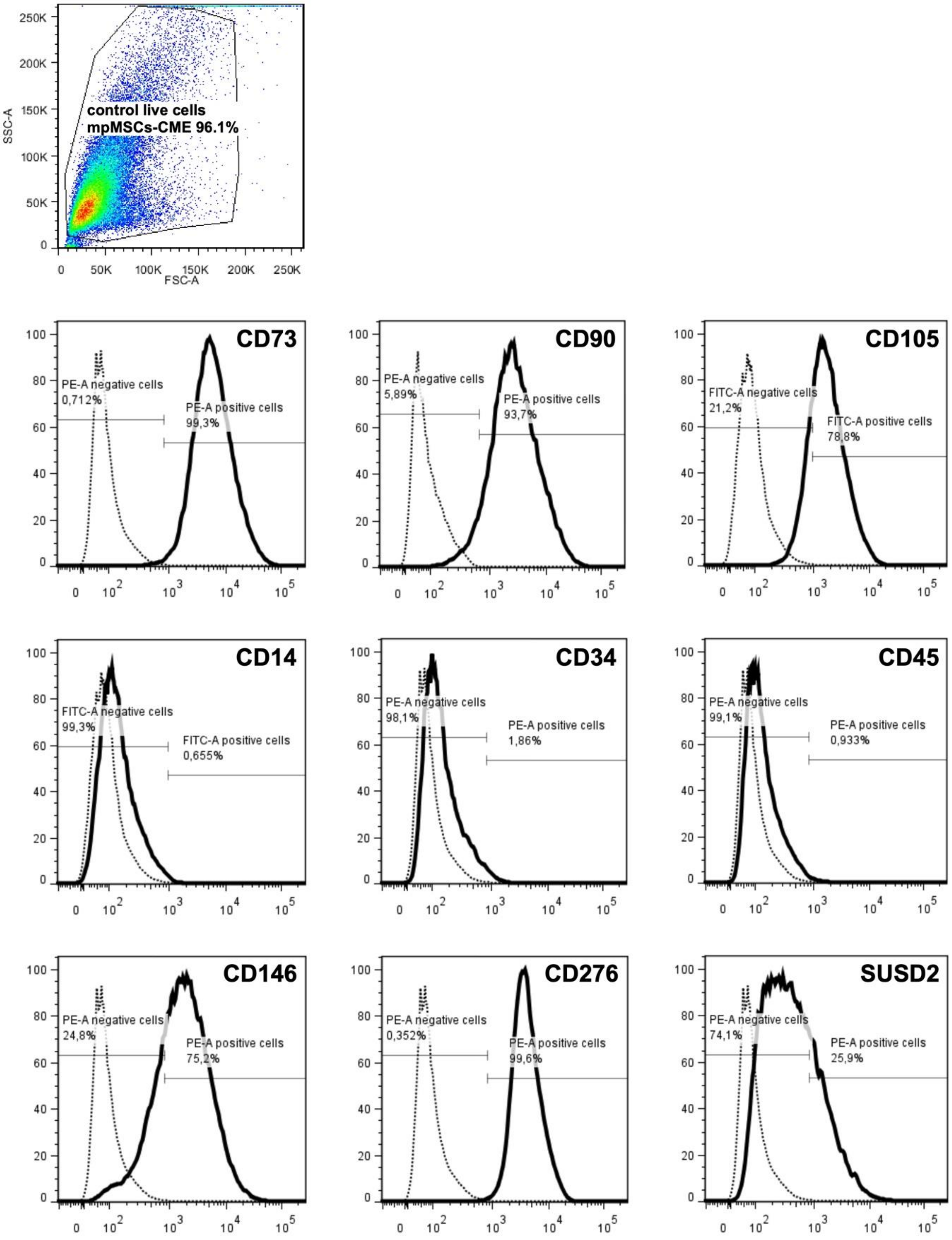

Online supplement Figure S8: Detection of cell surface markers on mpMSCs expanded for two passages in CME-medium.

The mpMSCs were expanded to the second passage of *in vitro* culture in CME-medium. The levels of cell surface antigen expression was investigated by FC. Live cells were gated by determining the side (SSC) and forward (FSC) scatters (upper left panel). The mean fluorescence intensities (x-axes) of the

different antigen stainings are presented as percent of maximum staining (y-axes) as indicated. All mpMSCs expanded in CME-medium expressed the mesenchymal antigens CD73 (99.3%) and CD90 (93.7%). Most cells expressed CD105 (78.8%), and less than 1.9% of cells expressed CD14, CD34, or CD45, respectively. The osteogenic marker CD146 (75.2%) and immune checkpoint antigen CD276 (99.6%) were prominent, and 25.9 % of cells expressed the stem cell marker SUSD2 at a moderate level. The figure shows a representative analysis of cells from the same placenta as presented in os Fig. 6.

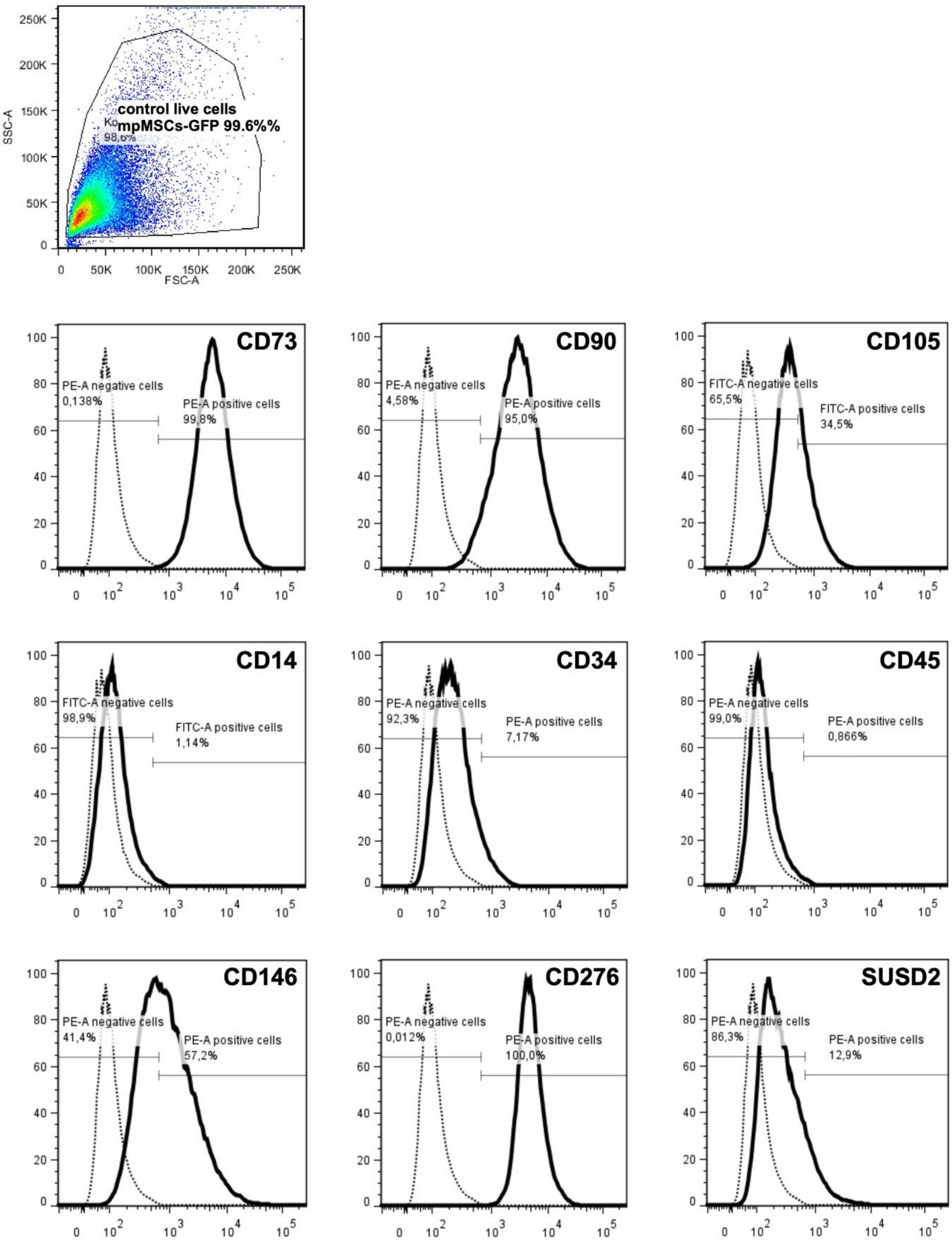

Online supplement Figure S9: Detection of cell surface markers on mpMSCs expanded for two passages in GMP-medium.

The mpMSCs were expanded *in vitro* to the second passage in GMP-medium. The levels of expression of cell surface antigens were investigated by FC. Live cells were gated by determining the side (SSC) and forward (FSC) scatters (upper left panel). The mean fluorescence intensities (x-axes) of the

different antigens staining are presented as percent of maximum staining (y-axes), as indicated. The mpMSCs expanded in GMP-medium expressed the mesenchymal antigens CD73 (99.9%) and CD90 (95%) at prominent levels. Only 34.5% of cells expressed moderate CD105, and CD105 staining intensity. CD14 (1.1%) was found not expressed while 7.17% of cells expressed CD34 with little staining intensity. Expression of CD45 was not recorded (0.87%). A moderate expression of the osteogenic marker CD146 was noted on 57.2% of cells. The immune checkpoint antigen CD276 was expressed on all mpMSCs (100%) with prominent staining intensity. The stem cell marker SUSD2 was expressed moderately on a few cells (12.9%). The figure shows a representative analysis of cells from the same placenta, as presented in os Fig. 6.

Online supplement tables:

Online supplement Table S1

**Antigen**	**antibody**	**clone**	**label**	**dilution**	**source**
**Primary antibodies:**					
CD73	moIgG1, k	AD2	ø	1:30	BioLegend
CD90	moIgG1	AS02	FITC	1:30	Dianova
CD105	moIgG1, k	266	ø	1:30	BD BioSciences
CD146	moIgG1	128018	PE	1:30	R&D Systems
CD276	rabbit IgG	ø	ø	1:2000	abcam
NG2	moIgG2a	LHM2	FITC	1:50	Santa Cruz Biotech
**Secondary antibodies:**					
Anti-rabbit Ig	goat	IgG	Cy3	1:300	Jackson Immunores.
Anti-mouse Ig	donkey	F(ab)	AlexaFluor488	1:50	BioLegend

Supplement Table 1: Primary antibodies against the antigens and secondary detection antibodies, label, dilution, and source as listed employed for immunofluorescence analyses of cryosections of human term placenta tissue. mo = mouse, k = κ light chain

Online supplement Table S2:

**Antigen**	**antibody**	**clone**	**label**	**dilution**	**source**
CD14	moIgG2a, k	M5E2	FITC	1:5	BD Pharmingen
CD34	moIgG1, k	581	PE	1:10	BioLegend
CD45	moIgG1, k	HI30	PE	1:6.25	Biolegend

CD73	moIgG1, k	AD2	PE	1:2.5	BD Pharmingen
CD90	moIgG2a	Thy1-A1	PE	1:5	R&D Systems
CD105	IgG1	SN6	FITC	1:5	BioRad
CD146	moIgG1	128018	PE	1:5	R&D Systems
CD276	moIgG1, k	MIH42	PE	1:20	BioLegend
SUSD2	mIgG1, k	W5C5	PE	1:10	Miltenyi Biotec

Supplement Table 2: Antibodies to the corresponding antigens, label, dilution, and source as listed used for FC of pMSCspMSCs. mo = mouse, k = κ light chain

Online supplement Table S3:

cDNA target	Upper	Lower
CD146	GAAGTCACCGTCCCTGTTTTC	CCCCGTTGTCGTTGGTTGT
CD276	TTTCCTTTCCCCTCCTTCCTCC	TGTGACCAGCACATGCTTCC
GAPDH	GAGTCAACGGATTTGGTCGT	TTGATTTTGGAGGGATCTCG
PPIAγ	TTCATCTGCACTGCCAAGAC	TCGAGTTGTCCACAGTCAGC

Supplement Table 3: Oligonucleotides employed for PCR of cDNAs to amplify the target genes as listed in 5′ to 3′ orientation.
